# Fusarioid community diversity associated with conifer seedlings in forest nurseries across the contiguous USA

**DOI:** 10.3389/fpls.2023.1104675

**Published:** 2023-01-25

**Authors:** J. T. Dobbs, M.-S. Kim, G. J. Reynolds, N. Wilhelmi, R. K. Dumroese, N. B. Klopfenstein, S. W. Fraedrich, M. M. Cram, J. Bronson, J. E. Stewart

**Affiliations:** ^1^ Colorado State University, Department of Agricultural Biology, Fort Collins, CO, United States; ^2^ USDA Forest Service, Pacific Northwest Research Station, Corvallis, OR, United States; ^3^ USDA Forest Service, Forest Health Protection – Region 3, Albuquerque, NM, United States; ^4^ USDA Forest Service, Forest Health Protection – Region 3, Flagstaff, AZ, United States; ^5^ USDA Forest Service, Rocky Mountain Research Station, Moscow, ID, United States; ^6^ USDA Forest Service, Southern Research Station, Athens, GA, United States; ^7^ USDA Forest Service, Forest Health Protection – Region 8, Athens, GA, United States; ^8^ USDA Forest Service, Forest Health Protection – Region 6, Medford, OR, United States

**Keywords:** damping off pathogens, haplotype diversity, conifer nurseries, geographic regions, *Fusarium*

## Abstract

**Introduction:**

Fusarioid fungi that cause damping-off and root diseases can result in significant losses to conifer crops produced in forest nurseries across the USA. These nurseries are vital to reforestation and forest restoration efforts. Understanding the diversity of Fusarioid fungi associated with damping-off and root diseases of conifer seedlings can provide an approach for targeted management techniques to limit seedling losses and pathogen spread to novel landscapes.

**Methods:**

This study identifies 26 *Fusarium* spp. (*F. acuminatum*, *F. annulatum*, *F. avenaceum*, *F. brachygibbosum*, *F. clavus*, *F. commune*, *F. cugenangense*, *F. diversisporum*, *F. elaeagni*, *F. elaeidis*, *F. flocciferum*, *F. fredkrugeri*, *F. fujikuroi*, *F. grosmichelii*, *F. ipomoeae*, *F. lactis*, *F. languescens*, *F. luffae*, *F. odoratissimum*, *F. oxysporum*, *F. queenslandicum*, *F. redolens*, *F. torulosum*, *F. triseptatum*, *F. vanleeuwenii*, & *F. verticillioides*), 15 potential species within *Fusarium* and *Neocosmospora* species complexes (two from *F. fujikuroi* species complex, nine from *F. oxysporum* species complex, three from *F. tricinctum* species complex, and one from *Neocosmospora* species complex), and four *Neocosmospora* spp. (*N. falciforme*, *N. metavorans*, *N. pisi*, & *N. solani*) and associated host information collected from conifer-producing nurseries across the contiguous USA.

**Results:**

Phylogenetic analyses identified Fusarioid fungi haplotypes that were associated with 1) host specificity, 2) localization to geographic regions, or 3) generalists found on multiple hosts across diverse geographic regions.

**Discussion:**

The haplotypes and novel species identified on conifer seedlings should be considered for further analysis to determine pathogenicity, pathogen spread, and assess management practices.

## Introduction

Forest nurseries produce seedlings of diverse conifer and hardwood species for timber production, forest restoration, and reforestation. In the United States (USA), 1.2 billion conifer seedlings are produced annually through container and/or bareroot stock types, primarily in the southern, northwestern, and north-central regions (Starkey et al., 2015a; Starkey et al., 2015b; Haase et al., 2020). However, nursery seedling production of both stock types for diverse purposes is frequently hampered by seedling diseases. Container nurseries tend to support higher seedling pathogen density, while bareroot nurseries tend to support greater pathogen species richness (Okorski et al., 2019). After transferring seedlings from nurseries to planting sites, seedling performance (i.e., survival and growth) is decreased when post-planting monitoring and treatments are lacking (Fargione et al., 2021). In addition, seedling survival after outplanting may be influenced by the introduction of nursery pathogens into the forest planting sites. For example, *Phytophthora* spp. can devastate hosts in natural stands after these pathogen species are carried on nursery stock and introduced into novel landscapes (Frankel et al., 2020). Once introduced, these pathogens are difficult to manage and can adversely impact tree planting programs and native vegetation surrounding planting sites.

Conifer seedlings are susceptible to a variety of soil- and seed-borne pathogens that can hinder production or have costly management implications due to the diversity in pathogen genera and species. Members of the genera *Cylindrocarpon* (syn: *Ilyonectria* & *Neonectria*), *Cylindrocladium* (syn: *Calonectria*), *Fusarium*, *Pythium*, *Phytophthora*, and *Rhizoctonia* have been found to cause damping-off and root disease (Dumroese et al., 2000; Cram, 2003; Dumroese and James, 2005; Vujanovic et al., 2007). Recently, *F. solani* species complex was placed in the genus *Neocosmospora* (Crous et al., 2021). Damping-off is the most common disease resulting in seedling wilting and mortality in North American forest nurseries, which primarily affects conifer seedlings within the first 4 to 6 weeks after germination (Cram, 2003). Symptoms of damping-off can range from wilting to pre- and post-emergent mortality of seedlings (Cram, 2003). If left untreated, damping-off and root disease pathogens can also cause root rot, stunting, and wilt in older seedlings (James et al., 1989; James et al. 1990b). Of the pathogens that cause seedling diseases, *Fusarium* spp., which are the most commonly isolated fungal pathogens from symptomatic seedlings, cause major economic losses and severe damage to conifer seedling production (James, 2004; Stewart et al., 2012).

Fusarioid fungi are very diverse and contain diverse pathogenic species that affect many important plant species for agriculture, silviculture, and horticulture worldwide, including grains, timber trees, and orchard trees (Marek et al., 2013; Boutigny et al., 2019; Dobbs et al., 2021). Several Fusarioid fungi have been isolated and identified from conifer seedlings, but not every identified species has been tested for pathogenicity on conifer hosts (James et al., 1989; James et al., 1990a; Stewart et al., 2012; Stewart et al., 2016). The most common Fusarioid fungi identified on conifer roots and in surrounding rhizosphere soil are *F. commune*, *F. oxysporum, F. proliferatum*, and *Neocosmospora solani*. In subsequent pathogenicity assays, isolates of these species have all been found to be pathogenic to the hosts from which they were isolated (James, 2005; Stewart et al., 2012). Virulence across strains within a species can vary, however, from non-pathogenic to highly virulent (Stewart et al., 2006).

Because morphology alone is insufficient to distinguish among strains of Fusarioid fungi that differ in virulence, molecular characterization or extensive assays are required to assess strain virulence. Further, molecular characterization of Fusarioid fungi is required because distinguishing morphological characteristics are rare or unreliable across some species (Stewart et al., 2012). Some species, such as *F. oxysporum*, have been found to contain host-specific formae speciales, but the full host range and level of specificity remains undetermined for many of these pathogenic strains (Edel-Hermann & Lecomte, 2019). The diversity observed within Fusarioid fungi inherently complicates their management, and lacking identification methods for pathogenic strains increases the risk of introducing these strains into novel landscapes. *Fusarium circinatum* is an economically and ecologically important canker pathogen that can spread easily from nurseries to planting sites through latent infections that decrease seedling establishment success and cause damage to mature trees in landscapes (Gordon et al., 2015). Unmitigated spread of these pathogens through anthropogenic means may increase the likelihood of pathogen adaptation to new hosts and landscapes, which could result in costly losses of seedlings in forestry landscapes and loss to existing forests (Corredor‐Moreno & Saunders, 2020; Fargione et al., 2021).

Our research aim was to assess the diversity of Fusarioid fungi associated with conifer-producing forest nurseries throughout the contiguous USA, based on DNA sequencing of two genes [translation elongation factor 1-α (*tef1α*) and RNA polymerase II second largest subunit (*rpb2*)]. We surveyed the nurseries to 1) identify patterns of geographic distribution and host range across Fusarioid fungi, and 2) determine if Fusarioid fungi haplotypes were widespread or isolated within a region.

## Materials and methods

### Sample and isolate collection

We made two collections of Fusarioid fungi. The first collection (from 2012 through 2016) was made across 11 states (representing federal, private, and state entities) throughout the western, midwestern, and southern USA (Stewart et al., 2020). For this collection, we visited container and bareroot forest nurseries and collected more than 300 isolates from soil, container substrates, and roots from asymptomatic and symptomatic conifer and non-conifer hosts; however, to increase the likelihood of identifying conifer pathogens, only 66 of those isolates that were collected from conifer host roots are reported in this study. Our second collection (from 2017 through 2020) targeted underrepresented conifer hosts and states identified after the first collection. Ten nurseries across eight states were surveyed about potential root disease problems. Based on their responses (**Supplemental Table 1**), five nurseries shipped conifer seedlings in 3.8-L, zip-lock-type bags on ice with the roots wrapped in damp paper towels to prevent desiccation. We visited three nurseries in 2021 and attempted to collect up to 20 seedlings (10 asymptomatic and 10 symptomatic) of each conifer species grown at each nursery. According to our survey agreements, specific-site information will remain confidential, and analyses were only conducted at the state level. The number of samples per site varied from 14 to 101 with 1 to 21 seedlings per host.

Fusarioid fungal isolates were obtained from plant samples by culturing root segments on Komada’s agar (Komada, 1976). Five root segments per seedling were surface disinfested in 10% commercial bleach for 2 minutes, washed twice in sterile water, and plated. After at least 3 days, when initial mycelial growth from root segments was observed, hyphal tips were transferred to ¼-strength potato dextrose agar (PDA; BD Difco™ Dehydrated Culture Media, Franklin Lakes, New Jersey, USA) in Petri dishes. Cultures were grown at 25°C for 14 days in the dark. Cultures were stored in the Stewart collection (Colorado State University) in glycerol broth at -80°C and/or on sterile filter paper stored at -20°C in coin cases with a desiccant to keep isolates dry.

**Figure 1:**
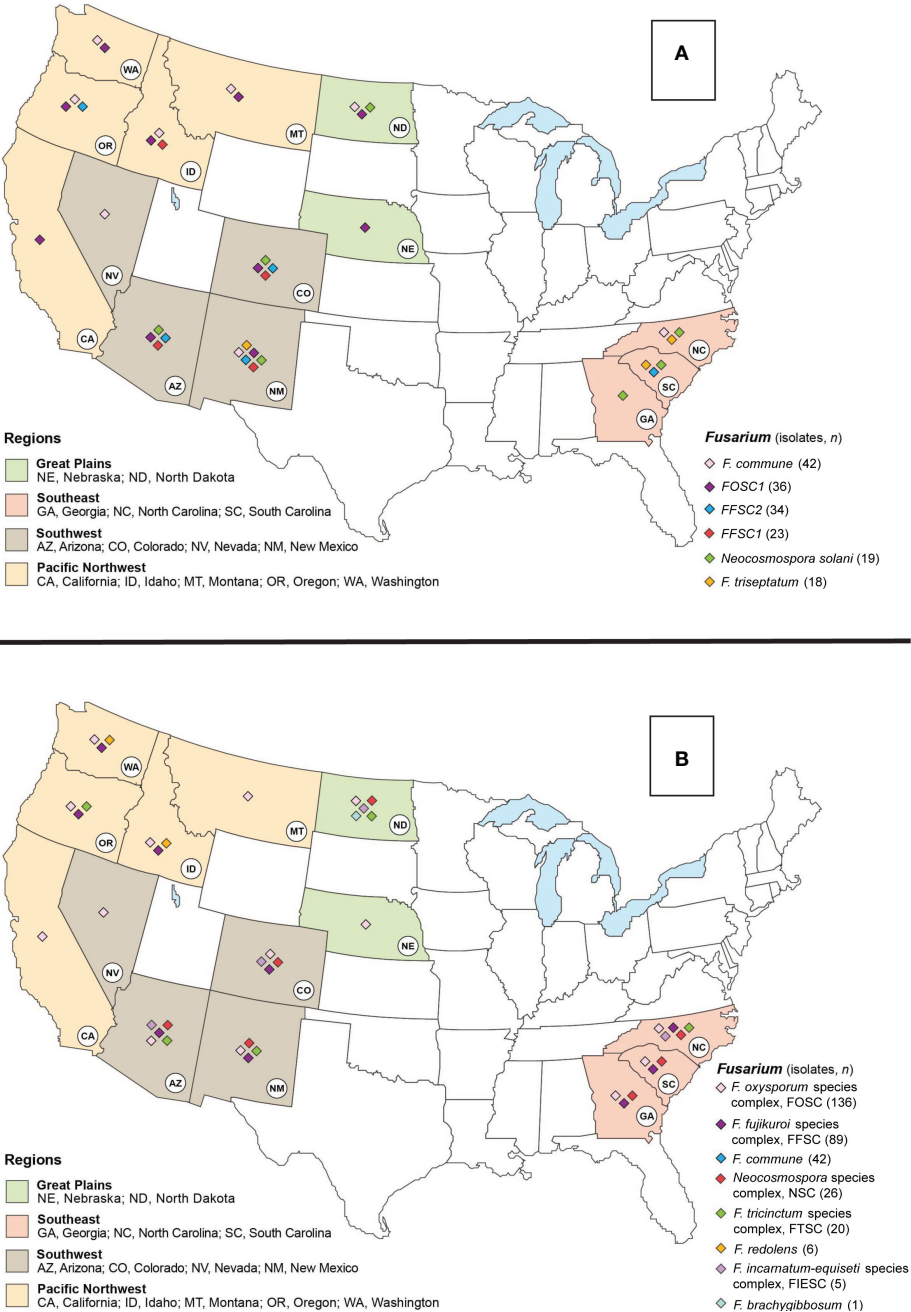
Map of the United States (USA) indicating the six most abundant Fusarioid fungi **(A)** and the Fusarioid species complexes **(B)** identified through a damping-off and root disease survey of conifer seedlings in states within the Southwest, Southeast, Pacific Northwest, and Great Plains regions. The colors on the states indicate their regional designation and the colored diamonds over the states indicates the presence of one of the six Fusarioid fungi identified in that state.

In total, we collected samples in 16 states (**Figure 1**) and obtained a total of 741 isolates from 27 host species. We selected 325 isolates collected from asymptomatic and symptomatic conifer hosts with associated tree species and state information for further analyses (these analyses excluded isolates from Michigan and Utah). A total of 66 isolates were obtained from the 2012-2016 collection and the remaining 259 isolates were from the 2017-2020 collection (**Supplemental Table 2**).

### DNA sequencing of *Fusarium* isolates

The 325 isolates were grown on ¼-strength PDA in Petri dishes and initially extracted using a Chelex100^®^ resin extraction protocol similar to Dobbs et al. (2021). Mycelia were scraped from cultures using a sterile spatula and transferred to 0.2-ml, 8-strip PCR tubes, followed by thermolyzation using a thermal cycler program of 100°C for 35 minutes and held at 4°C until ready for use in polymerase chain reaction (PCR). If poor quality DNA sequencing was obtained in the forward direction or low-quality DNA was obtained from the isolate, a CTAB extraction protocol from Dobbs et al. (2021) was used as an alternate DNA extraction method. Isolates were sequenced at two loci, *tef1α* (Primers EF-1: 5’-ATGGGTAAGGARGACAAGAC-3’ and EF-2: 5’-GGARGTACCAGTSATCATGTT-3’; O’Donnell et al., 1998) and *rpb2* (Primers RPB2-6F: 5’-TGGGGKWTGGTYTGYCCTGC-3’ and fRPB2-7cR: 5’-CCCATRGCTTGYTTRCCCAT-3’; Liu et al., 1999). Both loci were amplified using a PCR cycle program of 94°C for 2 min, 40 cycles of 94°C for 40s, 58°C for 40s, and 72°C for 30s, and 72°C for 5 min (Dobbs et al., 2020). Products were electrophoresed on a 1.5% agarose gel to visualize amplified PCR product using GelRed^®^ (Biotium). Amplified products were sequenced in both directions using Sanger sequencing at Eurofins Genomics (https://eurofinsgenomics.com/en/home/). As described in O’Donnell et al. (2021), sequences were checked visually for base-score quality using Geneious Prime v. 2022.0.1 (https://www.geneious.com/) and identified to putative species through BLAST analysis in the National Center for Biotechnology Information (NCBI) database (https://www.ncbi.nlm.nih.gov/), Fusarium-ID v. 3.0 (http://isolate.fusariumdb.org/blast.php), (O’Donnell et al., 2015; O’Donnell et al., 2021). Species were confirmed through phylogenetic analysis using DNA sequences from *Fusarium* spp. type strains obtained from GenBank links from the Fusarioid-ID database (Crous et al., 2021).

### Phylogenetic analysis

A Bayesian inference phylogeny was constructed from partitioned, concatenated *tef1α* and *rpb2* sequences. Sequences from representative Fusarioid fungi type strains were aligned to survey isolate sequences with MUSCLE sequence aligner using default settings (Edgar, 2004). Haplotypes were generated using DNAsp 6 (Rozas et al., 2017). IQtree2 v2.2.0 was used to generate the maximum likelihood bootstrap support values for the concatenated phylogeny from the haplotype file with 1000 pseudoreplicates, and Modelfinder was used to determine substitution model for the phylogeny (Minh et al., 2020). Substitution models for each locus were determined independently by partitioning. IQtree2 symmetry testing was conducted to ensure each partition did not reject the stationary and homogeneous sequence evolution assumptions (Naser-Khdour et al., 2019). Beauti2 was used to format the aligned sequences for subsequent use in BEAST2 to generate the Bayesian phylogeny (Bouckaert et al., 2019).

### Statistical analyses

All statistical analyses were conducted in R v. 4.0.2 (R Core Team, 2022). To visualize the distribution of potentially pathogenic haplotypes within Fusarioid fungi, relative abundance heatmaps of Fusarioid fungi by host and by state were generated from 325 isolates using the “gplots” and “BBmisc” packages (Bischl et al., 2017; Warnes et al., 2020).

To test the hypotheses that the composition of Fusarioid fungi communities differed among regions and between host genera, we conducted a permutational multivariate analysis of variance (PERMANOVA). Fusarioid fungi communities were grouped into four regions, 1) Pacific Northwest consisting of Idaho, Oregon, Washington, Montana, and California; 2) Southwest consisting of Colorado, New Mexico, Arizona, and Nevada; 3) Southeast consisting of Georgia, North Carolina, and South Carolina; and 4) Great Plains consisting of Nebraska and North Dakota (**Figure 1**). Community dissimilarity was contrasted by host genus and region using Bray-Curtis distances with a PERMANOVA using the ‘vegan’ package (Oksanen et al., 2020).

## Results

### Phylogenetic analyses

**Figure 2:**
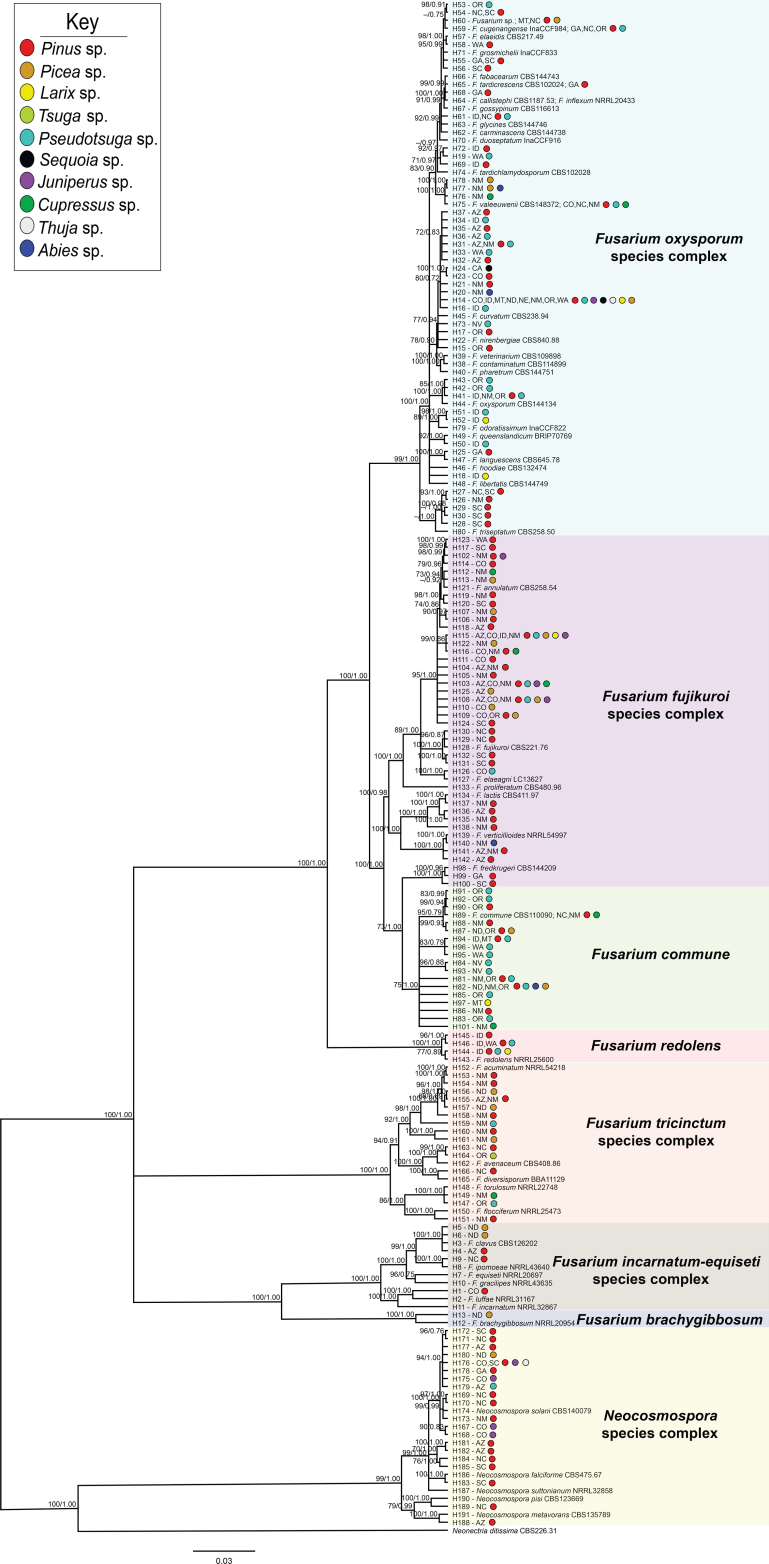
Bayesian posterior probability phylogeny inferred from concatenated *tef1α* and *rpb2* haplotypes from sequences of 325 Fusarioid fungi isolates collected from conifer hosts across the contiguous USA. Node values indicate maximum likelihood bootstrap support based on 1000 pseudo-replicates and Bayesian posterior probability values above 70%/0.75, respectively. The tree is rooted to *Neonectria ditissima* isolate sequences collected from GenBank (accession # *tef1α*: JF735783.1; *rpb2*: DQ789798.1). For each haplotype (denoted by H#), states are listed and colored circles indicate the host genera from which the isolates within that haplotype were collected. Fusarioid fungi and species complex haplotype clades are grouped together within colored boxes.

Each partition did not reject the stationary and homogeneous sequence evolution assumptions with p-values greater than 0.05 (*tef1α*: SymPval = 0.300, MarPval = 0.184, & IntPval = 0.495; *rpb2*: SymPval = 0.673, MarPval = 0.615, & IntPval =0.526). Phylogenies were constructed using sequences from the 325 isolates that included isolates that were sequenced at either or both the *tef1α* and *rpb2* loci (**Supplemental Table 2**). Sequences for these isolates were aligned and sorted into 191 haplotypes with *Neonectria ditissima*, a sister taxon to *Fusarium* (Geiser et al., 2021), collected from GenBank (accession # *tef1α*: JF735783.1; *rpb2*: DQ789798.1) serving as the outgroup (**Supplemental Table 2**). Of the eight *Fusarium* species complexes [*F. fujikuroi* species complex (FFSC); *F. incarnatum-equiseti* species complex (FIESC); *F. nisikadoi* species complex (FNSC); *F. oxysporum* species complex (FOSC); *F. redolens* species complex (FRSC); *F. sambucinum* species complex (FSAMSC); *Neocosmospora* species complex (NSC); *F. tricinctum* species complex (FTSC)], most formed well-supported clades, with all with bootstrap values and posterior probabilities above 75 and 0.80 in the maximum likelihood and Bayesian phylogenies, respectively (**Figure 2**). The majority of the haplotypes were collected from single hosts (85%; 124/146) and states (83%; 121/146). The remaining 15% (22/146) and 17% (25/146) of haplotypes were derived from two to seven host genera and two to eight states, respectively (48 haplotypes were derived from GenBank references only).

### Fusarioid species and relative abundance

**Figure 3:**
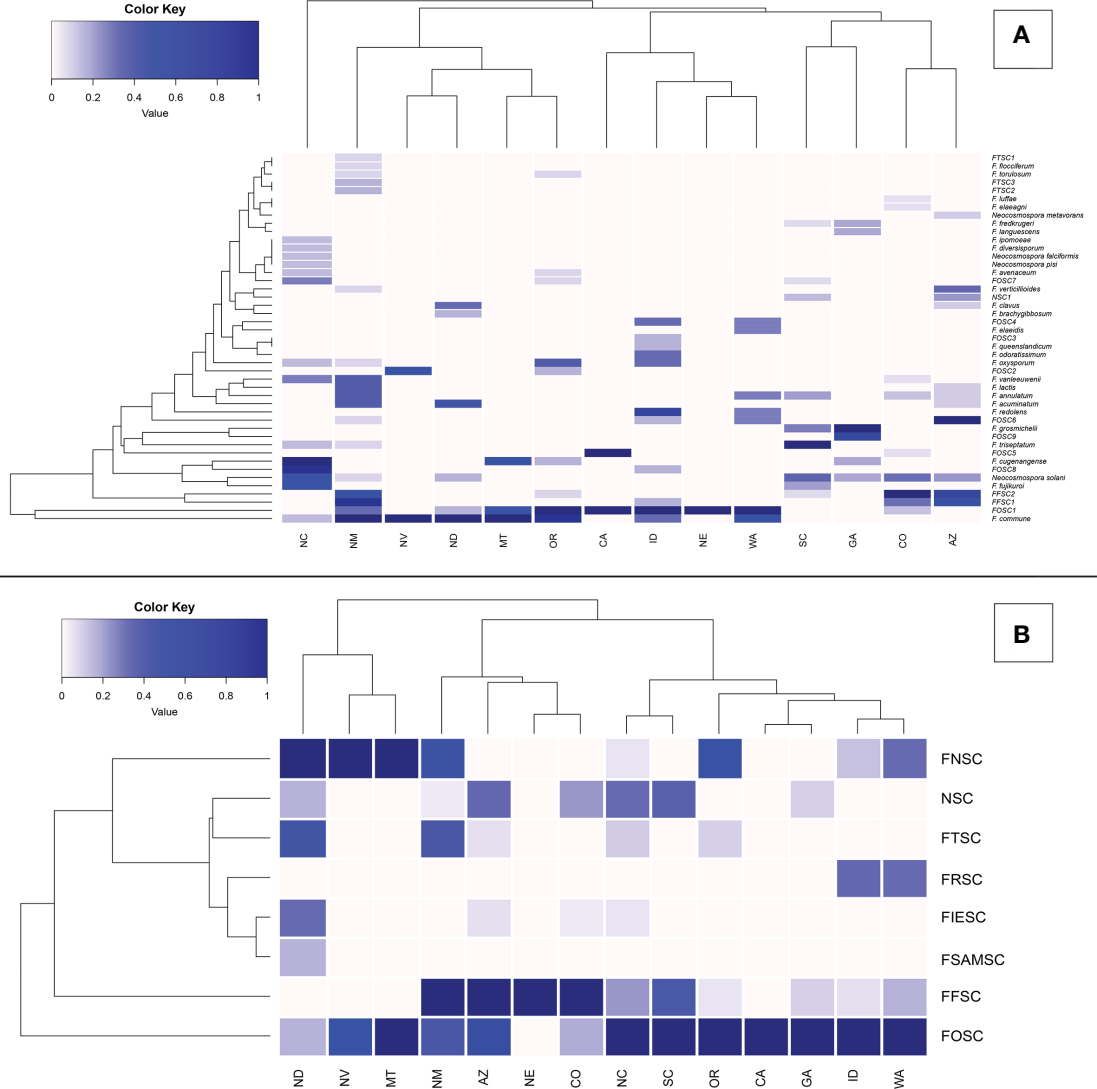
Relative abundance of Fusarioid fungi isolates **(A)** and Fusarioid species complexes **(B)** collected from conifer seedling host species from 14 states surveyed across the contiguous USA. Abbreviations for species complexes include FNSC - Fusarium nisikadoi species complex; NSC - Neocosmospora species complex; FTSC - Fusarium tricinctum species complex; FRSC - Fusarium redolens species complex; FIESC - Fusarium incarnatum-equiseti species complex; FSAMSC - Fusarium sambucinum species complex; FFSC - Fusarium fujikuroi species complex FOSC - Fusarium oxysporum species complex. Data were normalized to indicate the proportion of Fusarioid fungi isolates collected from each conifer host with mint-cream to green signifying the range of proportion from 0 to 1, respectively. Dendrogram shows the hierarchical relationship between each Fusarioid fungi (y-axis) and host (x-axis).

**Figure 4:**
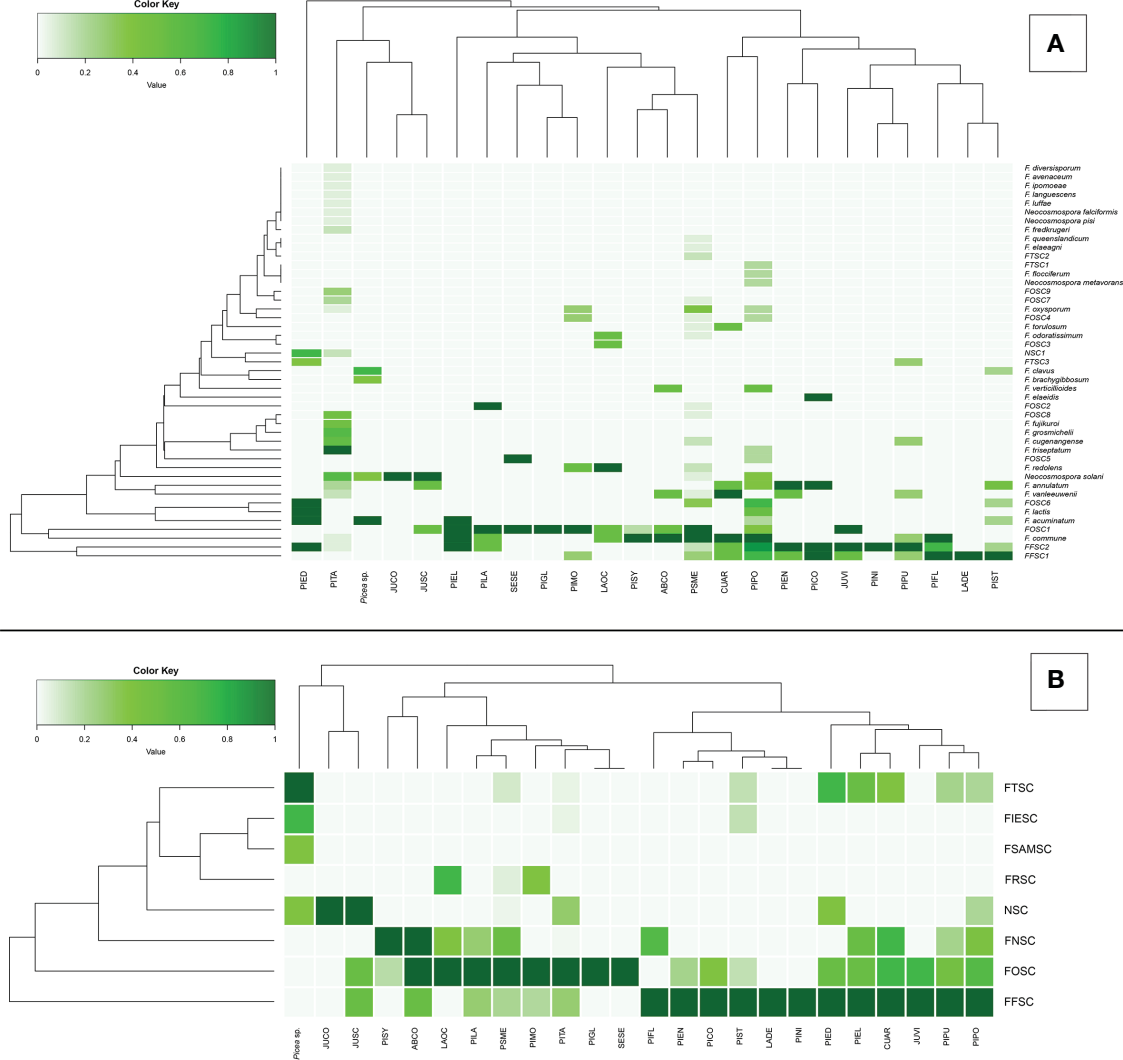
Principal coordinates analysis (PCoA) plots of Fusarioid fungi analyzed by the four regions [Pacific Northwest (PNW), Southwest (SW), Southeast (SE), and Great Plains (GP)] of the USA, where isolates were collected and the three most common host genera. Abbreviations for species complexes include FNSC - Fusarium nisikadoi species complex; NSC - Neocosmospora species complex; FTSC - Fusarium tricinctum species complex; FRSC - Fusarium redolens species complex; FIESC - Fusarium incarnatum-equiseti species complex; FSAMSC - Fusarium sambucinum species complex; FFSC - Fusarium fujikuroi species complexFOSC - Fusarium oxysporum species complex.

From 325 isolates with *tef1α* and/or *rpb2* sequences, a total of 26 *Fusarium* spp. were identified, and 15 potential *Fusarium* spp. were recognized that could not be assigned to the species level because only two loci (*tef1α* and *rpb2*) were sequenced. However, these 15 potential *Fusarium* spp. could all be assigned to four *Fusarium* species complexes [two from FFSC (*FFSC1*, *FFSC2*), nine from FOSC (*FOSC1*, *FOSC2*,…*FOSC9*), three from FTSC (*FTSC1*, *FTSC2*, *FTSC3*), and one from NSC (*NSC1*) members]. In addition, four *Neocosmospora* spp. were identified from the NSC (**Supplemental Tables 2**, **3**). Relative abundances of Fusarioid fungi were analyzed by state (**Figure 3A**) and host (seedling species) (**Figure 4A**). The most commonly collected species, in order of relative abundance, were *F.* commune, FOSC1, FFSC2, FFSC1, *Neocosmospora solani* (syn. *F. solani*), and *F. triseptatum*. Members within the FOSC and FFSC were the most commonly collected species (**Figures 3B**, **4B**).

Of these six species, *FOSC1* was collected from the most states, *F. triseptatum* from the least, with the other four species intermediate (**Table 1**). The number of plant species that were associated with each Fusariod fungal species followed the same general pattern, although *FFSC1* was associated with the highest number of plant species (**Table 1**).

**Table 1:** The six most commonly collected *Fusarium* and *Neocosmospora* spp. in order of abundance (left to right), the number of USA states in which they were observed, and the number (and percentage) of host species surveyed (n = 27) and the conifer genera from which they were isolated.

	*F. commune*	*FOSC1* ^a^	*FFSC2* ^b^	*FFSC1* ^c^	*N. solani* (syn. *F. solani*)	*F. triseptatum*
States (n)	8	9	5	4	7	2
Associations (n; %)	11; 40.7	12; 44.4	14; 51.8	11; 40.7	6; 22.2	2; 7.4
Genera						
*Abies*	X	X				
*Cupressus*	X		X	X		
*Juniperus*		X	X	X	X	
*Larix*	X	X		X		
*Picea*	X	X	X	X	X	
*Pinus*	X	X	X	X	X	X
*Pseudotsuga*	X	X	X	X	X	
*Thuja*		X			X	

^a^FOSC1, Fusarium oxysporum species complex 1.

^b^FFSC2, Fusarium fujikuroi species complex 2.

^C^FFSC1, Fusarium fujikuroi species complex 1.

### Fusarioid community variation

**Figure 5:**
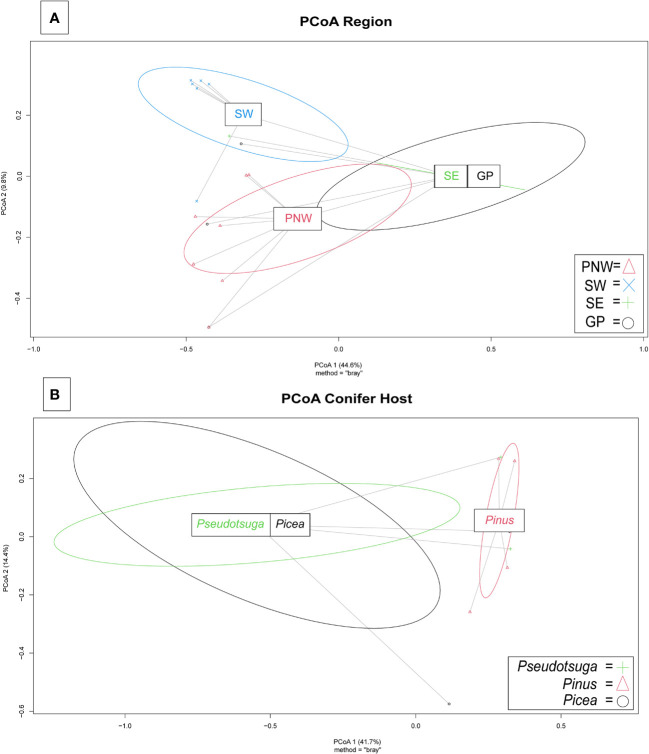
Principal coordinates analysis (PCoA) plots of Fusarioid fungi analyzed by the four regions [Pacific Northwest (PNW), Southwest (SW), Southeast (SE), and Great Plains (GP)] of the USA, where isolates were collected and the three most common host genera.

Variation among Fusarioid community structure among geographic regions was best described along two principal axes which explained 44.6% and 9.8% of the variation, respectively (**Figure 5A**). Variation among Fusarioid community structure among host genera was also best described along two principal axes which explained 41.7% and 14.4% of the variation, respectively (**Figure 5B**).

The PERMANOVA highlighted differences within the Fusarioid community that were associated with geographic region (R^2^ = 0.2197, F = 3.378, P = 0.003), but no distinct communities were found to be associated with the conifer host genus (R^2^ = 0.1853, F = 1.0236, P = 0.385). The Fusarioid communities identified in the Southeast and Great Plains regions were most similar (ordinated closer to one another) followed by the Southeast and Pacific Northwest (**Figure 5A**). The Southwest region was the most dissimilar (ordinated furthest away from other regions) region (**Figure 5A**). Fusarioid communities associated with *Pseudotsuga* and *Picea* were more similar to each other, while those associated with *Pinus* were the most distinct but still similar to the other host genera (**Figure 5B**).

## Discussion

In this study, we surveyed the diversity of Fusarioid fungi and strains in conifer-producing forest nurseries in relation to geographic range and host species, which provides a baseline toward understanding disease threats and invasive potential associated with these Fusarioid fungi and genetic strains. We identified 26 *Fusarium* spp., 14 potential *Fusarium* spp., one potential *Neocosmospora* sp., and four *Neocosmospora* spp. associated with conifers across the USA. Of these, several species (e.g., *F. annulatum*, *F. clavus*, *F. cugenangense*, *F. diversisporum*, *F. elaeagni*, *F. elaideidis*, *F. flocciferum*, *F. fredkrugeri*, *F. grosmichelii*, *F. inflexum*, *F. ipomoeae*, *F. lactis*, *F. languescens*, *F. odoratissimum*, *F. queenslandicum*, *F. torulosum*, *F. triseptatum*, *F. vanleeuwenii*, *N. falciforme*, *N. metavorans*, and *N. pisi*) have not previously been identified in association with conifer seedlings in North America. Further, since Fusarioid fungi been the subject of considerable taxonomic revision in recent years, not much is currently known about several of the species that were identified in our study. Our study highlights the need for further characterization and understanding of the role of these species as pathogens in conifer nurseries.

The importance of accurate phylogenetic analysis and maintaining genomic databases was highlighted in this study. as many Fusarioid fungi have been reclassified (e.g., *Neocosmospora solani* syn. *Fusarium solani*) (Šišić et al., 2018; Lombard et al., 2019; Xia et al., 2019; Crous et al., 2021; Yilmaz et al., 2021). Curated databases are also necessary for accurate species identification, such as Fusarium-ID (http://isolate.fusariumdb.org/blast.php) or Fusarioid-ID (Crous et al., 2021). Because these databases sometimes resulted in multiple species identifications for our isolates, we relied on phylogenetic analyses with type specimens included to help accurately identify our isolates at the species level (Crous et al., 2021). However, we found that molecular analyses reduce, but do not eliminate, the need for time-consuming morphological identification. Based on the two loci, *tef1α* and *rpb2*, we were not able to resolve all isolates at the species level. The use of diagnostic morphological features, although not always produced for all species (Stewart et al., 2006), may have further resolved some isolates to species.

Determining Fusarioid fungi composition and genetic variation of strains within species is an essential first step toward understanding the disease severity caused by Fusarioid fungi on conifer seedlings. Many Fusarioid fungi are morphologically similar and require molecular techniques to differentiate these cryptic species (Stewart et al., 2012). Variation in pathogenicity and virulence observed among cryptic species and other strains has management implications because some endophytic and saprophytic strains serve beneficial roles (e.g., Dumroese et al., 2012) that require no management, whereas others may be highly virulent pathogens to the plant of interest (Constantin et al., 2021). Furthermore, the characterization of Fusarioid fungi and genetically distinct strains is requisite for identifying potential movement of pathogenic strains between nurseries, states, and/or regions to prevent/limit further spread and reduce the impact of these strains on seedling production (Liebhold et al., 2012; Bate et al., 2016).

In this study, geographic region had a more significant influence on Fusarioid community compositions than the host genus. Even though seedling movement between nurseries was not tracked in this study, we did observe divergent community compositions of Fusarioid fungi that were differentiated by geographic and regional locations, suggesting that the movement of Fusarioid fungi and/or genotypes on seedlings and seed across regions may influence or shift Fusarioid fungi communities. In addition, the distinct Fusarioid fungi composition between the Pacific Northwest and Southwest regions, where we conducted site visits, could have been attributed to differences in site conditions and cultural practices that we observed. Management practices likely alter population densities of Fusarioid fungi and the frequency and severity of the diseases they cause. Members of the FOSC were found most prominently in this study, representing 42% (137/325) of the total isolates. *Fusarium oxysporum* has been recently divided and reclassified into several different species based on the genetic distinctiveness of isolates that belonged to many formae speciales (Lombard et al., 2019). *Fusarium oxysporum* has historically been noted as a pathogen on conifers (James et al., 1989; James, 2004; Stewart et al., 2012). However, pathogenic species on conifers within the newly described FOSC species have not yet been characterized, and this deserves further investigation as we found several new species associated with conifier in our study. We did not, however, conduct virulence assays in this study, which would be necessary to delineate pathogenic species of conifers within this species complex. We also identified isolates that belonged to several species complexes outside of the FOSC, including *F. fujikuroi* (27%; 89/325), *F. nisikadoi* (that contains *F. commune*) (13%; 41/325), *Neocosmospora* (8%; 26/325), *F. tricinctum* (6%; 20/325), and *F. redolens* (2%; 6/325). Members within these species complexes (e.g., *F. proliferatum*, *F. commune*, *Neocosmospora solani*, and *F. acuminatum*) have been found to be pathogenic to conifer species (James, 2004; Stewart et al., 2012; Stewart et al., 2016), though pathogenicity assays on conifers should be completed on newly named species for confirmation.

Several of the species identified in our study have been characterized as pathogens that cause root rot diseases on non-coniferous hosts. These pathogens may pose a potential hazard to landscape plantings as they may be adapted to native, non-conifer hosts surrounding planting sites. For examples, *F. flocciferum* causes root rot of pea (*Pisum sativum*) and faba bean (*Vicia faba*) (Šišić et al., 2020), *F. lactis* causes internal fruit rot of sweet pepper (*Capsicum anuum*) (Sekiguchi et al., 2021), and *F. torulosum* causes crown rot of wheat (*Triticum* sp.) (Karlsson et al., 2021). Previously, *F. inflexum* and *F. ipomoeae* were found as pathogens on non-coniferous hosts, causing wilt disease of beans (*Vicia faba*) (Schneider & Dalchow, 1975) and leaf spot disease of peanuts (*Arachis hypogaea*) (Xu et al., 2021), respectively. *Neocosmospora falciforme* was previously found in association with two *Pinus* spp., but it was not tested for pathogenicity (Herron et al., 2015). In addition, *F. fredkrugeri* is a newly described species that was isolated from rhizosphere soils of non-coniferous hosts, but this species has not been identified as a pathogen (Sandoval-Denis et al., 2018). Several Fusarioid fungi, *F. acuminatum*, *F. commune*, *F. oxysporum*, *F. proliferatum*, and *Neocosmospora solani* (syn. *F. solani*), are known to cause similar symptoms on commonly grown conifer species, including Douglas-fir (*Pseudotsuga menziesii*) and ponderosa pine (*Pinus ponderosa*) (Stewart et al., 2006; James & Dumroese, 2007). The shared evolutionary history of these damping-off and root rot pathogens has not been well studied and deserves further investigation. Although these Fusarioid fungi may work independently as pathogens, they may also function in concert with other Fusarioid fungi. Multiple weak pathogens may work in concert to overwhelm host defenses to cause the development of disease and associated symptoms (Abdullah et al., 2017). Microbial species compositions can influence the plant host’s ability to defend against pathogens (Vujanovic et al., 2007). The influence of Fusarioid fungi composition on host range and disease severity is, however, not well studied, and remains unknown for several newly characterized species, including *F. annulatum,* which was recently separated from *F. proliferatum*. It remains undetermined how Fusarioid fungi that are identified as pathogens on non-coniferous hosts may influence, either directly or indirectly, disease severity on coniferous seedlings and vice versa. Further investigations are needed to determine if shared virulence genes among these Fusarioid fungi contribute to pathogenicity on the same hosts.

Fusarioid fungi, such as *F. oxysporum*, and *Neocosmospora solani* (syn. *F. solani*), can transfer conditionally dispensable, lineage-specific chromosomes that may increase the competitiveness of receiving strains to cause disease on plant hosts (Dobbs et al., 2020; Ayukawa et al., 2021). Some *Fusarium* spp., such as *F. oxysporum* f. sp. *koae*, can cause death of older host trees if conditions become favorable for the pathogen and the host trees become stressed (Dobbs et al., 2020). With the increasing effects of climate change, higher temperatures may exacerbate tree stress and increase their susceptibility to opportunistic pathogens (Dumroese & James, 2005). The virulence of some weaker *Fusarium* spp. has been found to increase by raising temperatures from 20°C to 30°C (Huang & Kuhlman, 1990). The ever-increasing threat of more diverse pathogens on conifer seedlings amplifies the need for rapid identification of pathogenic *Fusarium* strains.

To accurately identify pathogenic Fusarioid fungi and strains, a better understanding of what determines and differentiates virulence among pathogenic species and strains within species is needed. Functional genomics analysis can provide a comprehensive understanding of damping-off/root rot pathogenesis of Fusaroid fungi. This genomics information can be further used to develop pathogen-specific primers for identification, detection, and monitoring. The haplotypes and novel species identified on conifers should be considered for further analysis to determine pathogen spread as it relates to management practices. The findings of this study emphasize the importance of mitigating spread of Fusarioid pathogens across regions and preventing introductions of genetically distinct pathogens into new landscapes.

## Data availability statement

The datasets presented in this study can be found in online repositories. The names of the repository/repositories and accession number(s) can be found in the article/**Supplementary Material**.

## Author contributions

JS and M-SK designed the study. JS, M-SK, GR secured funding. JD, GR, NW, JB, SF collected samples. JD and JB processed samples. JD analyzed the data. JD, JS, M-SK, NBK wrote the draft, and GR, NW, RD, MC, SF edited the paper. All authors contributed to the article and approved the submitted version.
