# Supplementary figures and images for: Fusarioid community diversity associated with conifer seedlings in forest nurseries across the contiguous USA
Source: Front Plant Sci. 2023 Jan 25;14:1104675. doi: 10.3389/fpls.2023.1104675 (PMC9930990; doi:10.3389/fpls.2023.1104675)

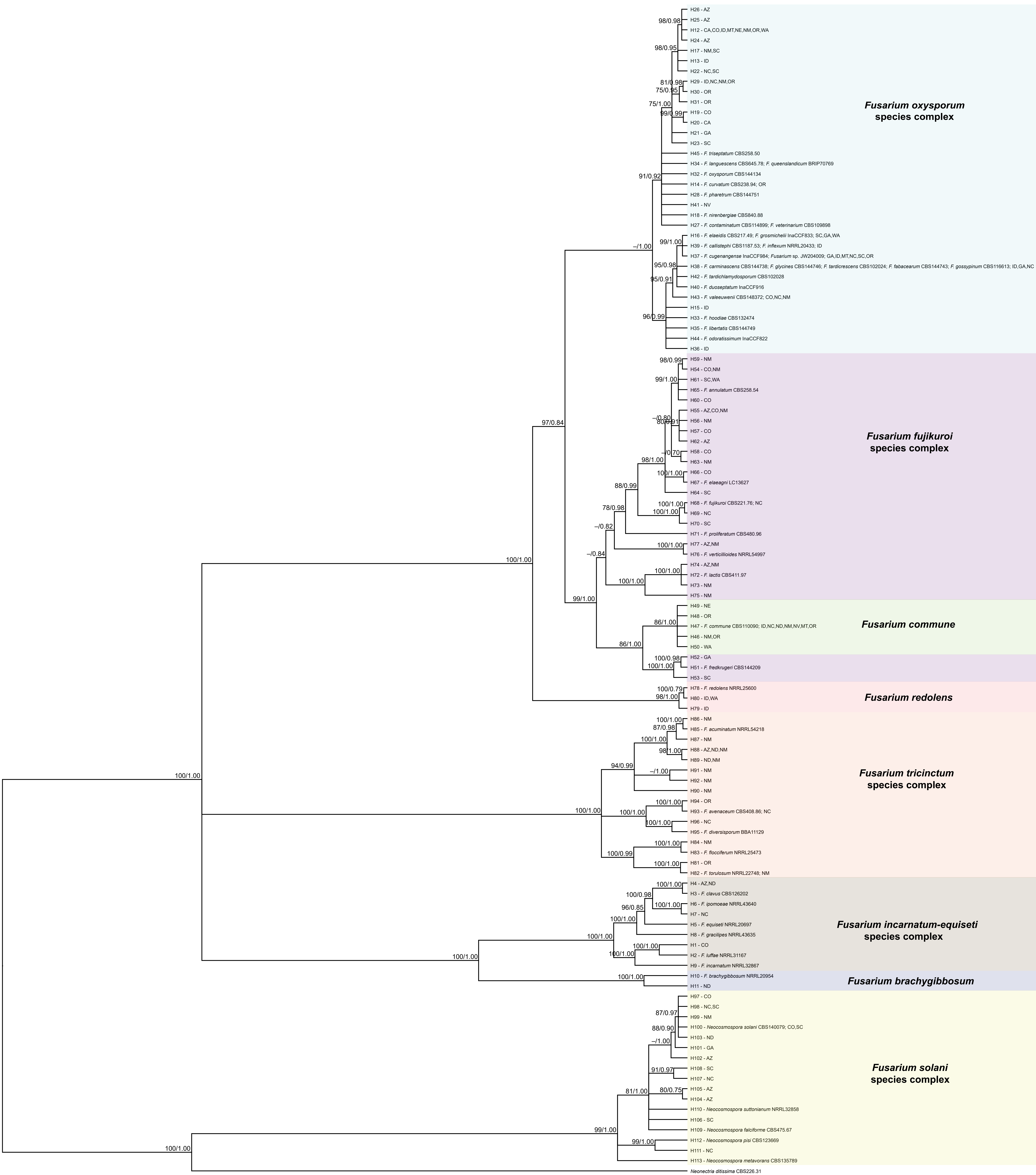

Supplement: Supplementary file 1 [file DataSheet_1.pdf]

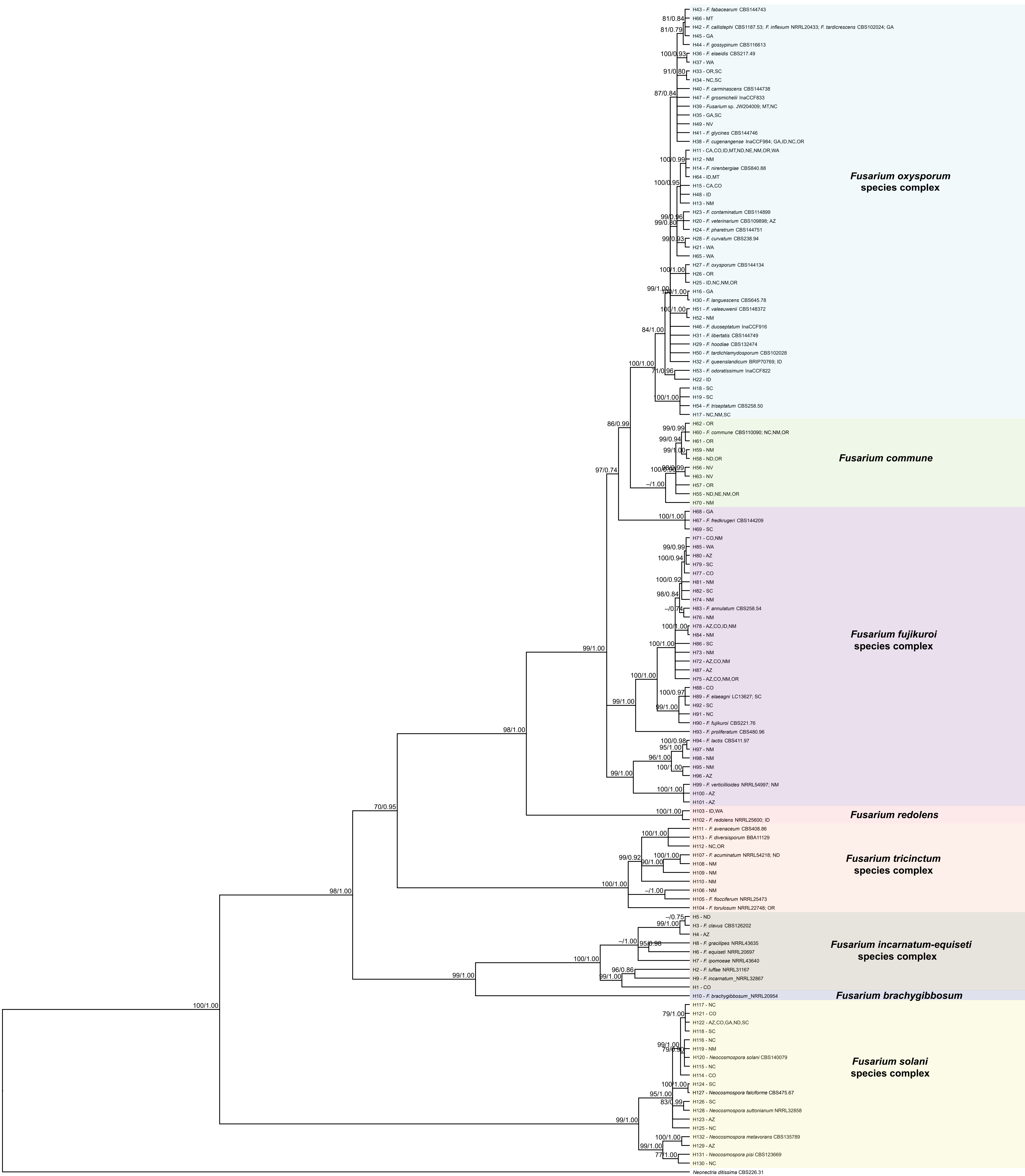

Supplement: Supplementary file 2 [file DataSheet_2.pdf]
